# Nature-Inspired Compounds: Synthesis and Antibacterial Susceptibility Testing of Eugenol Derivatives against *H. pylori* Strains

**DOI:** 10.3390/ph16091317

**Published:** 2023-09-18

**Authors:** Simone Carradori, Alessandra Ammazzalorso, Sofia Niccolai, Damiano Tanini, Ilaria D’Agostino, Francesco Melfi, Antonella Capperucci, Rossella Grande, Francesca Sisto

**Affiliations:** 1 Department of Pharmacy, “G. d’Annunzio” University of Chieti-Pescara, Via dei Vestini 31, 66100 Chieti, Italy; simone.carradori@unich.it (S.C.); francesco.melfi@unich.it (F.M.); rossella.grande@unich.it (R.G.); 2 Department of Chemistry “Ugo Schiff”, University of Florence, Via della Lastruccia 3–13, 50019 Sesto Fiorentino, Italy; sofia.niccolai@stud.unifi.it (S.N.); damiano.tanini@unifi.it (D.T.); antonella.capperucci@unifi.it (A.C.); 3 Department of Pharmacy, University of Pisa, Via Bonanno 6, 56126 Pisa, Italy; ilaria.dagostino@unipi.it; 4 Department of Biomedical, Surgical and Dental Sciences, University of Milan, Via Pascal 36, 20133 Milan, Italy; francesca.sisto@unimi.it

**Keywords:** eugenol, *Helicobacter pylori*, antibacterial, semisynthesis, chalcogens, MIC, MBC

## Abstract

The antimicrobial properties of one of the most important secondary metabolites, Eugenol (**EU**), inspired us to design and synthesize three different series of derivatives enhancing its parent compound’s anti-*Helicobacter pylori* activity. Thus, we prepared semisynthetic derivatives through (A) diazo aryl functionalization, (B) derivatization of the hydroxy group of **EU**, and (C) elongation of the allyl radical by incorporating a chalcogen atom. The antibacterial evaluation was performed on the reference NCTC 11637 strain and on three drug-resistant clinical isolates and the minimal inhibitory and bactericidal concentrations (MICs and MBCs) highlight the role of chalcogens in enhancing the antimicrobial activity (less than 4 µg/mL for some compounds) of the **EU** scaffold (32–64 µg/mL).

## 1. Introduction

Nature has always been a great source of medicaments; providing drugs directly or inspiring their design. Plants have only chemistry to fight for their survival from microbial attacks or weather and this is why they produce several bioactive molecules and metabolites that humans have studied and used for a wide variety of purposes, including the treatment of infectious diseases and cancer, in the preservation of food, and in cosmetics [1,2]. In fact, the World Health Organization (WHO) has estimated that 80% of people worldwide resort to medicinal plants for their primary health care [3].

**Figure 1:**
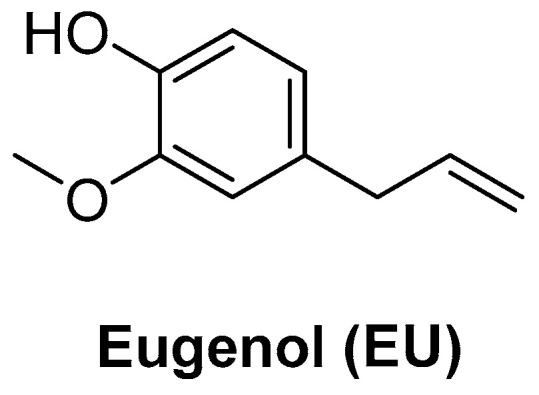
Chemical compound structure of eugenol.

In this context, essential oils are widely recognized as an interesting source of bioactive compounds that are worthy of investigation, especially for their phenolic components. This is due to their peculiar multitarget pharmacology, which is probably due to the presence and positioning of the hydroxyl function on the phenolic core [4]. Among these small molecules, eugenol (**EU**, chemically, 4-allyl-2-methoxyphenol, Figure 1) has emerged for its bioactivity and safety profile (with a dose of safety corresponding to 2.5 mg/Kg body) [5]. This has prompted the WHO to put it on its list of compounds that are generally recognized as safe (GRAS) [6].

Although it is abundant in cloves (*Syzygium aromaticum*), it can also be found in various plants, giving them its typical pleasant odor and taste. The high value of this compound and its derivatives in the medicinal chemistry field is justified by its multifaceted activities, including its broad-spectrum antibacterial [7,8], antioxidant [9,10], antifungal [11,12,13], antiviral [14,15,16] and antiprotozoal [17] properties, among its many others [18,19,20].

**EU** acts as a membrane active agent, causing a loss of membrane integrity of bacteria through several mechanisms of action, such as the inhibition of lipid peroxidation and ROS generation, cytoplasmatic and cell wall lysis, cell contents leakage, cytoplasm coagulation, impairment of membrane proteins and enzymatic activities, and the depletion of the proton motive force [21,22,23].

The presence of the phenolic group is widely recognized to be related to the overall antimicrobial and antibiofilm properties, since it confers a hydrophilic feature to the compound, allowing penetration into the lipopolysaccharide bilayer and entry in the cell, where it disrupts the membrane depolarization due to its proton exchange capability and the final cellular contents leakage and cell lysis. Additionally, the number and position of hydrocarbon substituents on the phenyl ring strongly impact the biological activity and should be considered in reliable drug design approaches. Infection of *H. pylori* is responsible for several gastric diseases, such as chronic gastritis, peptic and duodenal ulcers [24]; in addition, this pathogen has been well recognized as an important risk factor for the development of gastric adenocarcinoma, which occurs in 1–2% of infected patients. The increasing incidence of inflammation, gastric cancer, intestinal metaplasia, and other severe illnesses caused by *H. pylori* requires careful attention as well as increased medical efforts to counteract the pathogen infection [25].

In the fight against *H. pylori* infection, a limited number of approved drugs and impactful strategies are currently available, and the main therapies are based on the combination of proton pump inhibitors (PPIs), clarithromycin (CLR), and amoxicillin (AMX) or metronidazole (MTZ). However, the occurrence of antibiotic resistance is one of the most important factors responsible for the failure of *H. pylori* eradication therapy; in this view, the identification of different resistant phenotypes and the development of personalized treatments based on antibiotic susceptibility are becoming increasingly important [26,27].

With the alarming increase in bacterial resistance to antibiotics, especially CLR and MTZ, alternative and complementary strategies are under evaluation, including the use of natural compounds and nanotechnological formulations [28]. Along with other structurally related phenols (such as thymol and carvacrol) [29,30,31], **EU** has been identified as an interesting hit compound in the search for novel, nature-inspired agents useful in the treatment of *H. pylori* infections [32].

In this work, we report the development of three series of **EU** derivatives (A–C):

- A, a diazo function was added in the *ortho* position, to explore the chemical space near the phenolic group and keep the OH function unsubstituted (**1**–**7**, Table 1);
- B, the phenolic group of the **EU** was alkylated (**8**–**14**) or incorporated into a carbamate (**16**–**18**) or ester (**19** and **20**, Table 1) moiety;
- C, the allylic portion of the **EU** was replaced by a differently substituted tail, including an epoxide ring (**21**, Table 1), or alcohol chains (**22**) containing a chalcogen (**23**–**30**).

The designed compounds **1**–**30** were synthesized through straightforward and high-yielding synthetic pathways and then in vitro tested to assess the antibacterial susceptibility of four *H. pylori* strains, including a reference compound from the NCTC culture collection (NCTC 11637) and three drug-resistant clinical isolates (namely F1, 23, and F40/499) [33].

## 2. Results and Discussion

### 2.1. Chemistry

**Scheme 1:**
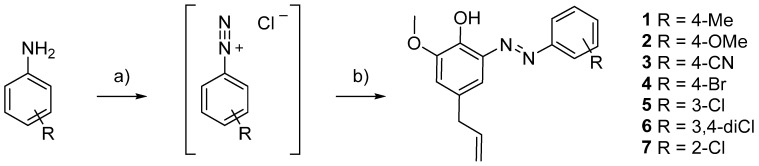
Synthetic route to azocompounds **1**–**7** (series A). Reagents and conditions: (a) NaNO_2_, 1.2 M HCl, 0 °C, 5 min; (b) **EU**, 1.32 M NaOH, 0 °C, 5 min.

Azoderivatives **1**–**7** were synthesized through the traditional diazotization reaction of the suitable substituted aniline with sodium nitrite in acid environment at 0 °C and the following addition of **EU** in basic solution for NaOH, as depicted in Scheme 1.

**Scheme 2:**
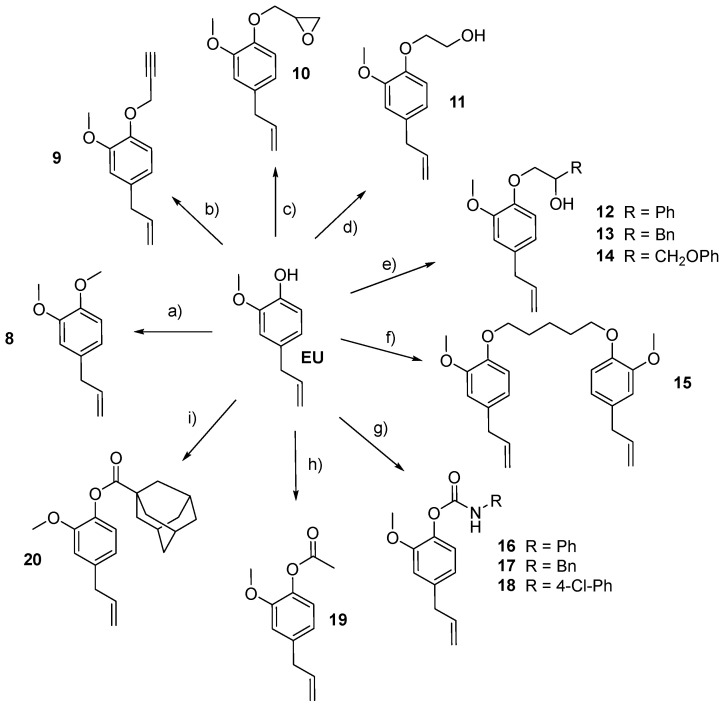
Synthetic routes to compounds **8**–**20** (series B). Reagents and conditions: (a) methyl iodide, ACN, reflux, 12 h; (b) propargyl bromide, K_2_CO_3_, ACN, reflux, 6 h; (c) epibromohydrin, K_2_CO_3_, ACN, reflux, 4 h; (d) 2-iodoethanol, K_2_CO_3_, DMF, 0 °C to reflux, 2 h; (e) suitable substituted epoxide, K_2_CO_3_, ACN, reflux, 12 h; (f) 1,5-dibromopentane, methanol, 70 °C, 12 h; (g) suitable isocyanate, triethylamine (TEA), dry ACN, r.t. to 60 °C, 24 h; (h) NaHCO_3_, Ac_2_O, r.t., 10 h; (i) 1-adamantanecarbonyl chloride, TEA, dimethylamino pyridine (DMAP), CH_2_Cl_2_, 0 °C to r.t., 48 h.

*O*-derivatization in series B to compounds **8**–**20** was performed as depicted in Scheme 2. In refluxing acetonitrile (ACN) and basic environment, **EU** was reacted with methyl iodide or propargyl bromide to obtain compounds **8** and **9**, respectively, or epibromohydrin for epoxide derivative **10**. The reaction of **EU** with 2-iodoethanol, in refluxing *N*,*N*-dimethylformamide (DMF), afforded the alcohol **11**; with proper epoxide—styrene oxide, 2,3-epoxypropyl benzene, or 1,2-epoxy-3-phenoxypropane—it gave derivatives **12**–**14**, respectively; with 1,5-dibromopentane in the suitable ratio yielding the dimeric ether **15**. Instead, the preparations of carbamates **16**–**18** were carried out by reacting **EU** with the proper isocyanates—phenyl isocyanate, benzyl isocyanate, or 4-chlorophenyl isocyanate, respectively. Esters **19** and **20** were synthesized by working in an excess of acetic anhydride or in the presence of 1-adamantanecarbonyl chloride, respectively.

**Scheme 3:**
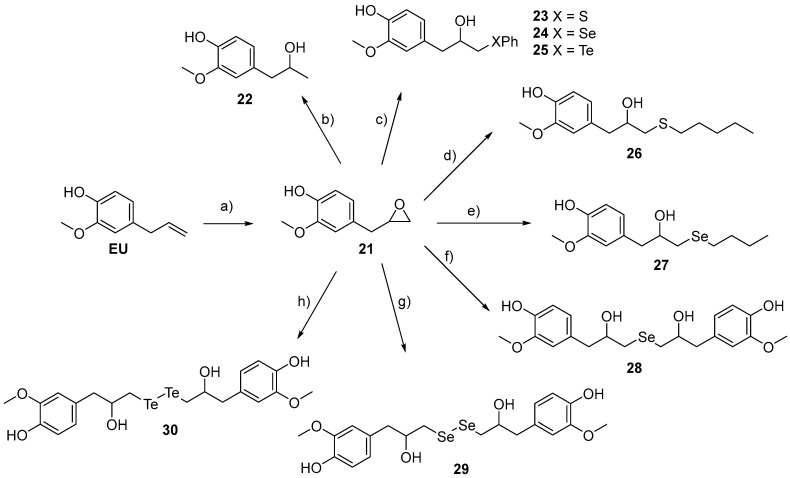
Synthesis of the **EU**-derived epoxide **21** and its derivatives **22**–**30** (series C). Reagents and conditions: (a) 3-chloroperbenzoic acid, CH_2_Cl_2_, 0 °C to r.t., 48 h; (b) LiAlH_4_, dry THF, reflux, 5 h; (c) PhXSiMe_3_ (X = S, Se, Te), TBAF, THF, r.t., 5 h; (d) 1-pentanethiol, Cs_2_CO_3_, TBAI, DMF, 0 °C to r.t., 5 h; (e) dibutyl diselenide, NaBH_4_, EtOH, 0 °C to r.t., 6 h; (f) in situ Na_2_Se (elemental selenium, rongalite, NaOH, H_2_O, r.t., 30 min), H_2_O, r.t., 12 h; (g) (Me_3_Si)_2_Se, TBAF, THF, r.t., 5 h; (h) in situ Na_2_Te (elemental tellurium, rongalite, NaOH, H_2_O, 90 °C, 2 h), H_2_O, 90 °C to r.t., 4 h.

The obtainment of series C chalcogen-containing derivatives is reported in Scheme 3. Epoxide **21** was easily prepared upon the reaction of **EU** with 3-chloroperbenzoic acid (mCPBA) in CH_2_Cl_2_ at room temperature (r.t.) for 48 h [34]. The alcohol **22** was synthesized following a literature-reported route via reduction of **21** with LiAlH_4_ in tetrahydrofuran (THF) [35,36]. Epoxide **21** was, then, employed as the common key precursor for the synthesis of the series of chalcogen-containing **EU** derivatives **23**–**30**. β-Phenylthio, β-phenylseleno, and β-phenyltelluro alcohols **23**–**25** were prepared via a ring-opening reaction of **21** with phenylchalcogenosylanes (PhSSiMe_3_, PhSeSiMe_3_, and PhTeSiMe_3_) under tetrabutylammonium fluoride (TBAF) catalysis [37]. The β-hydroxy sulfide **26** was obtained upon treatment of **21** with 1-pentanethiol in the presence of the Cs_2_CO_3_/tetrabutylammonium iodide (TBAI) system using DMF as a solvent [38]. Similarly, the β-hydroxy selenide **27** was synthesized through the ring-opening of **21** with butaneselenolate anions, easily generated in situ by reductive cleavage of dibutyl diselenide with NaBH_4_ in ethanol (EtOH) [39]. The bidentate selenide **28** was prepared through a related, on-water ring-opening procedure, using sodium selenide (Na_2_Se) as the nucleophilic species [40]. Na_2_Se was generated in situ from elemental selenium using rongalite (sodium hydroxymethanesulfinate dihydrate) as the reducing agent and H_2_O as the solvent. The synthesis of the diselenide **29** was pursued by exploiting the TBAF-catalyzed, silicon-mediated reaction of **21** with bis(trimethylsilyl)selenide [(Me_3_Si)_2_Se]. The ring-opening reaction of epoxide **21** also enabled the synthesis of β-hydroxy ditelluride **30** following our recently reported rongalite-mediated methodology [41]. All of the nucleophilic ring-opening reactions herein reported occurred with high regioselectivity, leading to the formation of the product as a result of the attack of the nucleophile onto the less hindered carbon atom of the epoxide **21**. In the case of compounds **28**–**30**, a mixture of diastereoisomers was achieved.

### 2.2. In Vitro Antibacterial Activity Studies against H. pylori

For this study, we used four strains of *H. pylori*, the commercial strain NCTC 11637 and three clinical isolates (F1, 23, F40/499), which have previously been identified by Gram staining and characterized in terms of catalase, urease, and oxidase activity. The antibiotic susceptibility was then undertaken with metronidazole (MTZ), clarithromycin (CLR), and amoxicillin (AMX) as benchmarks. The assessment of genotypic differences among sensitive and resistant clinical isolates should allow better phenotypic discrimination against resistant strains, which usually affect the susceptibility data not only of the parent compound, but also of its derivatives. In the literature, it is also reported that the resistance to MTZ (NCTC 11637 and F40/499) is usually highly associated with the mutational inactivation of the gene encoding an oxygen-independent NADPH nitroreductase, whereas resistance to CLR (F1 and F40/499) is related to the mutation in the macrolide target. The antibacterial activity data for series A–C compounds are reported in Table 1 and comprise minimal inhibitory concentration (MIC) and minimal bactericidal concentration (MBC) values.

These compounds are endowed with low molecular weights and small chemical structures that allow the design of further derivatives through a wide panel of chemical modifications and derivatization aimed at exploring structure–activity relationships (SARs), modulating their bioactivity and overcoming their poor water solubility and volatility, and obtaining derivatives with improved pharmacokinetic profiles.

Observing Table 1, one can see that **EU** exhibited MIC values between 32 and 64 µg/mL and that MBC values were higher (64–128 µg/mL). A comparable anti-*H. pylori* activity against the four strains can be noticed, thus suggesting a mechanism of action not related to their resistance pathways for this compound. In any case, the efficacy of **EU** seemed to be suitable against MTZ-resistant strains.

In series A, electron-donating groups on the phenyl ring in the *para* position, such as the methyl group in compound **1** and the methoxy function in **2**, seem to be slightly tolerated, resulting in increased MIC values in which the latter are more potent than the former, exhibiting MIC = 32 µg/mL and MBC values between 32 and 64 µg/mL. Instead, the presence of electron-withdrawing groups in the phenyl *para*-position, such as cyano (**3**) or bromine (**4**), and the presence of two chlorine atoms in *meta* and *para* (**6**) led to a dramatic decrease in activity. In the end, *m*-chloro (**5**) and *o*-chloro (**7**) phenyl derivatives showed the best results in the series, with MIC values of 8 µg/mL and MBC values between 8 and 16 µg/mL on all the tested strains. Despite chlorine atom and methyl group showing similar van der Waals radii and lipophilic properties, their different electronic features differently affect the antibacterial activity.

In series B, small substituents such as the methyl (**8**), propargyl (**9**), and methylenepoxide (**10**) groups did not significantly affect the bioactivity of the compounds. Interestingly, compound **11**, containing a primary hydroxy function, showed a low inhibitory potency, while **12**–**14**, also bearing a hydroxy function on the aliphatic chain, along with carbamate **17**, are the best compounds of the series in terms of antibacterial activity. Observing their structures, we could assume that the presence of a phenyl ring contributes to an optimal hydrophobic/polar balance and, thus, the antibacterial profile of such compounds. As regards derivative **15**, the presence of two **EU** units linked through a flexible *O*-pentyl spacer resulted in being detrimental to the antibacterial activity against the NCTC 11637 and 23 strains, while a better profile than **EU** is highlighted against F1 and F40/499 strains. The other two carbamates, **16** and **18**, are not worthy of interest, exerting an intermediate activity. However, the presence of chloride in the *para* position of the phenyl ring in **18** led to a 1-dilution-fold decrease in both MIC and MBC values with respect to the unsubstituted phenyl compound **17**. In the end, esters such as acetate (**19**) and the bulkier adamantyl ester (**20**) were not able to achieve interesting MIC values.

Notably, series C compounds exhibited the greatest results in the inhibition of *H. pylori* growth. The epoxide and epoxide-opened ring in **21** and **22**, respectively, worsened the antibacterial profile of **EU**. Compound **24**, containing a selenium nucleus, showed the best activity on the MTZ-resistant F40/499 strain, with MIC and MBC values equal to 2 µg/mL, while its analogues with sulfur (**23**) and tellurium (**25**) atoms were found to be less potent on the strains panel (MICs = 8–16 µg/mL), though still remaining highly promising antibacterial agents. The replacement of the phenyl ring of **23**–**24** with aliphatic (pentyl) chains on the chalcogen atom, leading to derivatives **26**–**27**, caused a decrease in antibacterial potency, with the sulfur compound **26** 1-dilution-fold more potent than the corresponding selenium analogue **27** on all of the tested strains. The structural complication of the chain in compounds **28**–**30** gave different results, with **29** being the most potent compound of the C series having MIC and MBC values that range from 2 to 4 µg/mL. However, when the Se–Se bond of **29** is replaced by a single Se atom (**28**) or a Te–Te bond (**30**), a decrease in activity was observed. These last modifications were attempts to keep the OH of the parent compound unsubstituted. The design of chalcogen-containing compounds in the C series is envisaged by the increasing interest in organochalcogen derivatives [42]. In fact, sulfur compounds are widely employed in medicinal chemistry as antibiotics or synthetic intermediates. Organoselenium compounds were found to possess a potent electrophilic activity, responsible for the attack on cysteine residues in proteins, especially antioxidative enzymes, resulting in their association with oxidative stress through thiol depletion, reactive oxygen species (ROS) overproduction, DNA damage, and mitochondrial dysfunctions [43]. Instead, tellurium organocompounds remain poorly investigated [44].

## 3. Materials and Methods

### 3.1. Chemistry

**General chemistry.** All commercially available chemicals and solvents were used as purchased. Anhydrous reactions were performed in flame-dried glassware after three cycles of vacuum/dry nitrogen and were run under a positive pressure of dry nitrogen. Chromatographic separations were performed on columns packed with silica gel (230–400 mesh, for flash technique). Reaction monitoring was performed through thin-layer chromatography (TLC) by using 0.2-mm-thick silica gel–aluminum-backed plates (60 F254). TLC spot visualization was performed under short and long wavelength (254 and 365 nm, respectively) ultra-violet irradiation and stained with ninhydrin or basic permanganate stains. ^1^H and ^13^C NMR were recorded on a spectrometer operating at 300/400 and 75/100 MHz, respectively. Spectra are reported in parts per million (*δ* scale) and internally referenced to the CDCl_3_, CD_3_OD, and DMSO-*d*_6_ signal, respectively, at *δ* 7.26, 3.31, and 2.50 ppm. Chemical shifts for carbon are reported in parts per million (*δ* scale) and referenced to the carbon resonances of the solvent (CDCl_3_ at *δ* 77.0, CD_3_OD at *δ* 49.0, and DMSO-*d*_6_ at *δ* 39.0 ppm). Data are shown as follows: chemical shift, multiplicity (s = singlet, d = doublet, t = triplet, q = quartet, qi = quintet, m = multiplet and/or multiplet resonances, br = broad signal, ap = apparent), integration and coupling constants (*J*) in Hertz (Hz). The ^1^H and ^13^C spectra confirmed the anticipated number of hydrogens and carbons for each compound, respectively. Melting points were measured on a Stuart^®^ melting point apparatus SMP1 (Fisher Scientific Italia, Segrate (MI), Italy) and are uncorrected (temperatures are reported in °C). Elemental analyses for C, H, and N were recorded on a Perkin-Elmer 240 B microanalyzer and the analytical results are within ± 0.4% of the theoretical values for all compounds. Mass spectra were recorded by electrospray ionization (ESI) using a Thermo LCQ-Fleet instrument. Mass spectra (LCMS (ESI)) were acquired using an Agilent 1100 LC-MSD VL system (G1946C, Agilent Technologies Italia Spa, Rome, Italy) by direct injection with a 0.4 mL/min flow rate using a binary solvent system of 95/5 MeOH/H_2_O. Samples were prepared in MeOH or DMSO/MeOH, according to the solubility properties of the test compound, in some cases in presence of formic acid. Mass spectra were performed in positive mode scanning over the mass range 100–1500 *m*/*z*, using a variable fragmentor voltage of 0–70 V.

Compounds **21** [33], **22** [45], **29** [40], and **30** [41] were prepared according to the literature. Spectroscopic data matched those reported.

#### Procedure and Characterization Data for Derivatives **1**–**30**

***General Procedure for the Synthesis of Compounds*** **1–7**

An aqueous solution of sodium nitrite (638.1 mg, 9.9 mmol in 5 mL of H_2_O) was added to an acidic solution (conc. HCl/H_2_O, 1/10 mL) of the proper substituted aniline (6.6 mmol) at 0–5 °C. The diazonium salt was then coupled by adding a mixture of **EU** (1.0 mL, 6.6 mmol) in an aqueous solution of NaOH (264.0 mg, 6.6 mmol in 5 mL of H_2_O) keeping the pH value at 8–9. Once the reaction was completed, the mixture was poured into H_2_O and extracted with CHCl_3_ three times (3 × 25 mL). The combined organic layers were dried over Na_2_SO_4_, filtered, and evaporated under reduced pressure to afford the crude products. These were then subjected to column chromatography on silica gel, with different *n*-hexane/EtOAc mixtures as eluent.

***(E)-4-allyl-2-methoxy-6-(p-tolyldiazenyl)phenol*** ***(*1*)***

Red-brown solid, m.p. 96–97 °C, 68% yield. Characterization data are in agreement with the previous literature [46].

***(E)-4-allyl-2-methoxy-6-[(4-methoxyphenyl)diazenyl]phenol (*2*)***

Red-brown solid, m.p. 98–99 °C, 78% yield. Characterization data are in agreement with the previous literature [46].

***(E)-4-[(5-allyl-2-hydroxy-3-methoxyphenyl)diazenyl]benzonitrile (*3*)***

Brown solid, m.p. 162–163 °C, 86% yield. ^1^H NMR (300 MHz, CDCl_3_) *δ* 3.44 (d, *J* 7.2 Hz, 2H, CH_2_), 3.94 (s, 3H, OCH_3_), 5.12–5.18 (m, 2H, =CH_2_), 5.94–6.07 (m, 1H, CH=), 6.85 (d, *J* 1.8 Hz, 1H, Ar), 7.39 (d, *J* 1.8 Hz, 1H, Ar), 7.81 (d, *J* 8.7 Hz, 2H, Ar), 7.94 (d, *J* 8.7 Hz, 2H, Ar), 12.84 (bs, 1H, ArOH). ^13^C NMR (75 MHz, CDCl_3_) *δ* 39.5, 56.5, 113.8, 116.5, 116.7, 118.3, 122.5, 124.0, 131.3, 133.4, 136.8, 137.3, 142.8, 148.9, 152.5. Anal. Calcd for C_17_H_15_N_3_O_2_: C, 69.61; H, 5.15; N, 14.33. Found: C, 69.58; H, 5.17; N, 14.37.

***(E)-4-allyl-2-((4-bromophenyl)diazenyl)-6-methoxyphenol (*4*)***

Red-brown solid, m.p. 123–124 °C, 77% yield. ^1^H NMR (300 MHz, CDCl_3_) *δ* 3.42 (d, *J* 6.6 Hz, 2H, CH_2_), 3.93 (s, 3H, OCH_3_), 5.11–5.17 (m, 2H, =CH_2_), 5.94–6.07 (m, 1H, CH=), 6.82 (d, *J* 1.8 Hz, 1H, Ar), 7.39 (s, 1H, Ar), 7.64 (d, *J* 8.7 Hz, 2H, Ar), 7.73 (d, *J* 8.7 Hz, 2H, Ar), 12.90 (bs, 1H, ArOH). ^13^C NMR (75 MHz, CDCl_3_) *δ* 39.6, 56.5, 155.9, 116.3, 123.6, 123.9, 125.5, 130.9, 132.6, 137.1, 141.6. Anal. Calcd for C_15_H_13_BrN_2_O: C, 56.80; H, 4.13; N, 8.83. Found: C, 56.85; H, 4.14; N, 8.87.

***(E)-4-allyl-2-[(3-chlorophenyl)diazenyl]-6-methoxyphenol (*5*)***

Brown solid, m.p. 83–84 °C, 93% yield. ^1^H NMR (300 MHz, CDCl_3_) *δ* 3.42 (d, *J* 7.2 Hz, 2H, CH_2_), 3.93 (s, 3H, OCH_3_), 5.11–5.18 (m, 2H, =CH_2_), 5.96–6.05 (m, 1H, CH=), 6.83 (d, *J* 2.4 Hz, 1H, Ar), 7.40–7.46 (m, 3H, Ar), 7.73–7.76 (m, 1H, Ar), 7.86 (t, *J* 2.0 Hz, 1H, Ar). ^13^C NMR (75 MHz, CDCl_3_) *δ* 39.6, 56.5, 116.1, 116.3, 121.0, 121.8, 124.0, 130.4, 130.8, 131.0, 135.5, 137.0, 141.7, 148.7. Anal. Calcd for C_16_H_15_ClN_2_O_2_: C, 63.47; H, 4.99; N, 9.25. Found: C, 63.44; H, 5.00; N, 9.29.

***(E)-4-allyl-2-[(3,4-dichlorophenyl)diazenyl]-6-methoxyphenol (*6*)***

Red solid, m.p. 147–148 °C, 87% yield. ^1^H NMR (300 MHz, CDCl_3_) *δ* 3.43 (d, *J* 7.2 Hz, 2H, CH_2_), 3.93 (s, 3H, OCH_3_), 5.12–5.18 (m, 2H, =CH_2_), 5.94–6.07 (m, 1H, CH=), 6.84 (d, *J* 1.8 Hz, 1H, Ar), 7.39 (d, *J* 1.2 Hz, 1H, Ar), 7.60 (d, *J* 8.7 Hz, 1H, Ar), 7.70 (d, *J* 1.8 Hz, 1H, Ar), 7.73 (d, *J* 2.1 Hz, 1H, Ar), 7.97 (d, *J* 2.4 Hz, 1H, Ar), 12.59 (bs, 1H, ArOH). ^13^C NMR (75 MHz, CDCl_3_) *δ* 39.5, 56.5, 116.3, 116.4, 122.2, 123.0, 122.9, 131.1, 133.9, 135.0, 136.9, 137.0, 141.6, 149.2. Anal. Calcd for C_16_H_14_Cl_2_N_2_O_2_: C, 56.99; H, 4.18; N, 8.31. Found: C, 56.96; H, 4.20; N, 8.26.

***(E)-4-allyl-2-[(2-chlorophenyl)diazenyl]-6-methoxyphenol (*7*)***

Red-brown liquid, 83% yield. ^1^H NMR (300 MHz, CDCl_3_) *δ* 3.43 (d, *J* 6.6 Hz, 2H, CH_2_), 3.94 (s, 3H, OCH_3_), 5.11–5.18 (m, 2H, =CH_2_), 5.95–6.08 (m, 1H, CH=), 6.83 (d, *J* 1.8 Hz, 1H, Ar), 7.38–7.42 (m, 3H, Ar), 7.56–7.58 (m, 1H, Ar), 7.90–7.93 (m, 1H, Ar), 13.39 (bs, 1H, ArOH). ^13^C NMR (75 MHz, CDCl_3_) *δ* 39.6, 56.4, 115.9, 116.3, 117.2, 124.3, 127.6, 130.5, 130.7, 131.9, 134.1, 137.1, 137.5, 142.0, 149.0. Anal. Calcd for C_16_H_15_ClN_2_O_2_: C, 63.47; H, 4.99; N, 9.25. Found: C, 63.50; H, 5.01; N, 9.28.

***General procedure for the synthesis of *8* and *9****

Potassium carbonate (552.8 mg, 4.0 mmol) was added to a mixture of **EU** (154 µL, 1.0 mmol) and the appropriate alkyl halide (1.5 mmol, methyl iodide for **8** and propargyl bromide for **9**) in refluxing ACN (0.4 M). The reaction mixture was diluted with H_2_O and extracted with CHCl_3_ (3 × 25 mL); the combined organic layers were dried over Na_2_SO_4_, filtered, and evaporated under reduced pressure. Purification by chromatography column on silica gel allowed the obtainment of the desired products, using different *n*-hexane/EtOAc mixtures as mobile phase.

***4-Allyl-1,2-dimethoxybenzene (*8*)***

Colorless oil, 71% yield. Characterization data are in agreement with the previous literature [47].

***4-Allyl-2-methoxy-1-(prop-2-yn-1-yloxy)benzene (*9*)***

Colorless liquid, 86% yield. Characterization data are in agreement with the previous literature [48].

***Synthesis of 2-[(4-allyl-2-methoxyphenoxy)methyl]oxirane (*10*)***

To a stirred solution of **EU** (462 µL, 3 mmol) in ACN (20 mL), K_2_CO_3_ (622 mg, 4.5 mmol) and epibromohydrin (250 µL, 3 mmol) were added, and the mixture was refluxed for 4 h. After dilution with EtOAc, the organic phase was extracted thrice with H_2_O, dried over Na_2_SO_4_, filtered, and concentrated under reduced pressure to afford the crude product. Purification by chromatography on silica gel (eluent cyclohexane/EtOAc 9:1) furnished the desired compound. Colorless oil, 66% yield. Characterization data are in agreement with the previous literature [49]. LCMS (ESI+) *m/z* = 221.1 [M + H]^+^; 243.1 [M + Na]^+^; 463.2 [2M + Na]^+^.

***Synthesis of 2-(4-allyl-2-methoxyphenoxy)ethanol (*11*)***

K_2_CO_3_ (346 mg, 2.5 mmol) and **EU** (154 µL, 1 mmol) were solubilized in DMF (15 mL) and allowed to stir for 1 h at room temperature. Then, 2-iodoethanol (156 µL, 2 mmol) was added and the reaction mixture was refluxed for 2 h. After cooling, the solvent was evaporated under reduced pressure. The residue was dissolved in CH_2_Cl_2_ and washed three times with distilled H_2_O. The organic phase was dried over Na_2_SO_4_, filtered, and concentrated under reduced pressure. The crude product was purified by chromatography on silica gel (eluent CHCl_3_) to obtain the desired compound. Yellow oil, 71% yield. ^1^H NMR (300 MHz, CDCl_3_) *δ* 3.33 (d, *J* 7.2 Hz, 2H), 3.85 (s, 3H), 3.89 and 4.10 (both t, *J* 5.1 Hz, 2H), 5.05 (s, 1H), 5.09 (d, *J* 9.0 Hz, 1H), 5.88–6.01 (m, 1H), 6.72 (m, 2H), 6.86 (d, *J* 9.0 Hz, 1H). ^13^C NMR (75 MHz, CDCl_3_) *δ* 39.8, 55.7, 61.3, 71.9, 112.3, 115.6, 115.7, 120.7, 134.2, 137.4, 146.2, 149.9. Anal. Calcd for C_12_H_16_O_3_: C, 69.21; H, 7.74. Found: C, 69.31; H, 7.73. LCMS (ESI+) *m/z* = 231.1 [M + Na]^+^; 453.2 [2M + Na]^+^.

***General procedure for the synthesis of alcohols *12*–*14****

K_2_CO_3_ (276 mg, 2 mmol) and **EU** (169 µL, 1.1 mmol) were added to a stirred solution of proper epoxide (styrene oxide, 2,3-epoxypropyl benzene, or 1,2-epoxy-3-phenoxypropane, 1 mmol) in ACN (15 mL). After 12 h of reflux, the reaction mixture was diluted with EtOAc, extracted thrice with H_2_O, dried over Na_2_SO_4_, filtered, and concentrated under reduced pressure. Crude products were purified by chromatography on silica gel (eluent: CH_2_Cl_2_/MeOH 95:5) to obtain the desired compounds.

***2-(4-Allyl-2-methoxyphenoxy)-1-phenylethanol (*12*)***

Pale yellow oil, 68% yield. ^1^H NMR (300 MHz, CDCl_3_) *δ* 3.35 (d, *J* 6.9 Hz, 2H), 3.86 (s, 3H), 3.98 (d, *J* 10.2 Hz, 1H), 4.16 (dd, *J* 10.2, 3.0 Hz, 1H), 5.04–5.13 (m, 3H), 5.89–6.04 (m, 1H), 6.71–6.75 (m, 2H), 6.87 (d, *J* 8.4 Hz, 1H), 7.29–7.46 (m, 5H). ^13^C NMR (75 MHz, CDCl_3_) *δ* 39.8, 55.8, 72.3, 76.5, 112.3, 115.8, 115.9, 120.8, 126.3, 127.9, 128.4, 134.4, 137.4, 139.6, 146.2, 149.9. Anal. Calcd for C_18_H_20_O_3_: C, 76.03; H, 7.09. Found: C, 76.12; H, 7.07. LCMS (ESI+) *m*/*z* = 307.1 [M + Na]^+^; 591.2 [2M + Na]^+^.

***1-(4-Allyl-2-methoxyphenoxy)-3-phenylpropan-2-ol (*13*)***

Yellow-orange oil, 72% yield. ^1^H NMR (300 MHz, CDCl_3_) *δ* 2.87–2.90 (m, 2H), 3.33 (d, *J* 6.6, 2H), 3.83 (s, 3H), 3.86–4.03 (m, 2H), 4.15–4.23 (m, 1H), 5.04–5.10 (m, 2H), 5.88–6.01 (m, 1H), 6.69 (s and d, 2H), 6.82 (d, *J* 8.1 Hz, 1H), 7.22–7.33 (m, 5H). ^13^C NMR (75 MHz, CDCl_3_) *δ* 39.5, 39.8, 55.8, 70.9, 74.2, 112.4, 115.7, 115.8, 120.7, 126.4, 128.4, 129.3, 134.3, 137.4, 137.7, 146.4, 149.9. Anal. Calcd for C_19_H_22_O_3_: C, 76.48; H, 7.43. Found: C, 76.33; H, 7.44. LCMS (ESI+) *m*/*z* = 321.1 [M + Na]^+^; 619.3 [2M + Na]^+^.

***1-(4-Allyl-2-methoxyphenoxy)-3-phenoxypropan-2-ol (*14*)***

Pale yellow oil, 65% yield. ^1^H NMR (300 MHz, CDCl_3_) *δ* 3.34 (d, *J* 6.6, 2H), 3.84 (s, 3H), 4.11–4.23 (m, 4H), 4.35–4.41 (m, 1H), 5.05–5.12 (m, 2H), 5.89–6.01 (m, 1H), 6.71–6.73 (m, 2H), 6.88–6.97 (m, 4H), 7.25–7.31 (m, 2H). ^13^C NMR (75 MHz, CDCl_3_) *δ* 39.8, 55.8, 68.5, 68.7, 71.6, 112.4, 114.5, 115.6, 115.7, 120.7, 121.1, 129.4, 134.3, 137.4, 146.3, 149.8, 158.4. Anal. Calcd for C_19_H_22_O_4_: C, 72.59; H, 7.05. Found: C, 72.68; H, 7.03. LCMS (ESI+) *m*/*z* = 315.1 [M + H]^+^; 337.1 [M + Na]^+^.

***Synthesis of 1,5-bis(4-allyl-2-methoxyphenoxy)pentane (*15*)***

**EU** (308 µL, 2.0 mmol) was added to a mixture of 1,5-dibromopentane (70 µL, 1.0 mmol) and K_2_CO_3_ (552.8 mg, 4.0 mmol) in MeOH (150 mL), at 70 °C for 12 h. After evaporation, H_2_O (25 mL) was added, and the crude was extracted with CHCl_3_ (3 × 25 mL). The combined organic layers were then dried over Na_2_SO_4_, filtered, and evaporated under reduced pressure. The crude product was purified through column chromatography on silica gel, using *n*-hexane/EtOAc 9:1 as eluent. Orange solid, m.p. 62–64 °C, 87% yield. ^1^H NMR (300 MHz, CDCl_3_) *δ* 1.60–1.68 (m, 2H, CH_2_CH_2_CH_2_), 1.89 (qi, *J* 6.8 Hz, 4H, CH_2_CH_2_CH_2_), 3.32 (d, *J* 6.7 Hz, 4H, CH_2_Ar), 3.83 (s, 6H, OCH_3_), 4.00 (t, *J* 6.8, 4H, OCH_2_), 5.02–5.11 (m, 4H, =CH_2_), 5.95 (ddt, *J* 16.8, 10.1, 6.7 Hz, 2H, Ar), 6.69 (m, 4H, Ar), 6.79 (d, *J* 7.8 Hz, 2H, Ar). ^13^C NMR (75 MHz, CDCl_3_) *δ* 22.6, 29.1, 29.8, 39.9, 56.0, 69.1, 112.5, 113.4, 115.7, 120.6, 132.9, 137.8, 147.0, 149.5. Anal. Calcd for C_25_H_32_O_4_: C 75.73; H, 8.13. Found: C 75.80; H, 8.15. LCMS (ESI+) *m*/*z* = 419.2 [M + Na]^+^.

***General procedure for the synthesis of carbamates *16*–*18****

TEA (209 µL, 1.5 mmol) was added to a solution of **EU** (230 µL, 1.5 mmol) in dry ACN (150 mL) at r.t. under a nitrogen atmosphere. The mixture was stirred for 15 min, then the proper isocyanate (phenyl isocyanate, *p*-chlorophenyl isocyanate, or benzyl isocyanate, 1.65 mmol) was added portionwise. The reaction mixture was stirred at 60 °C for 24 h, it was then cooled down to room temperature. The mixture was diluted with EtOAc, washed with distilled H_2_O and then dried over Na_2_SO_4_, it was then filtered, and evaporated under reduced pressure. The crude products were purified by chromatography on silica gel using CHCl_3_ as the eluent.

***4-Allyl-2-methoxyphenyl phenylcarbamate (*16*)***

White solid, m.p. 98–100 °C, 57% yield. ^1^H NMR (300 MHz, CDCl_3_) *δ* 3.39 (d, *J* 6.9 Hz, 2H), 3.83 (s, 3H), 5.08 (s, 1H), 5.13 (d, *J* 9.3 Hz, 1H), 5.90–6.03 (m, 1H), 6.79 (d, *J* 9.6 Hz, 2H), 7.08 (m, 3H), 7.31 (t, *J* 7.8 Hz, 2H), 7.44 (d, *J* 8.1 Hz, 2H). ^13^C NMR (75 MHz, CDCl_3_) *δ* 40.1, 55.8, 112.7, 116.1, 118.5, 120.6, 123.0, 123.6, 129.0, 137.0, 137.5, 139.0, 151.3. Anal. Calcd for C_17_H_17_NO_3_: C, 72.07; H, 6.05; N, 4.94. Found: C, 71.98; H, 6.06; N, 4.97. LCMS (ESI+) *m*/*z* = 306.1 [M + Na]^+^; 589.2 [2M + Na]^+^.

***4-Allyl-2-methoxyphenyl benzylcarbamate (*17*)***

White solid, m.p. 77–79 °C, 72% yield. ^1^H NMR (300 MHz, CDCl_3_) *δ* 3.37 (d, *J* 6.6 Hz, 2H), 3.83 (s, 3H), 4.45 (d, *J* 6.0 Hz, 2H), 5.08 (s, 1H), 5.12 (d, *J* 9.0 Hz, 1H), 5.41 (bs, 1H), 5.89–6.02 (m, 1H), 6.76 (d, *J* 9.3 Hz, 2H), 7.03 (d, *J* 7.5 Hz, 1H), 7.29–7.36 (m, 5H). ^13^C NMR (75 MHz, CDCl_3_) *δ* 40.0, 45.3, 55.8, 112.7, 116.0, 120.6, 122.9, 127.5, 127.6, 128.6, 137.1, 138.2, 138.6, 151.4, 154.6. Anal. Calcd for C_18_H_19_NO_3_: C, 72.71; H, 6.44; N, 4.71. Found: C, 72.59; H, 6.45; N, 4.73. LCMS (ESI+) *m*/*z* = 320.1 [M + Na]^+^; 617.2 [2M + Na]^+^.

***4-Allyl-2-methoxyphenyl (4-chlorophenyl)carbamate (*18*)***

Colorless crystals, 60% yield; m.p. 124–126 °C. ^1^H NMR (300 MHz, CDCl_3_) *δ* 3.38 (d, *J* 7.2 Hz, 2H), 3.83 (s, 3H), 5.08 (s, 1H), 5.13 (d, *J* 9.3 Hz, 1H), 5.89–6.01 (m, 1H), 6.79 (d, *J* 9.6 Hz, 2H), 7.05 (d, *J* 7.5 Hz, 2H), 7.25–7.29 (m, 3H), 7.39 (d, *J* 8.7 Hz, 2H). ^13^C NMR (75 MHz, CDCl_3_) *δ* 40.0, 55.8, 112.7, 116.2, 119.7, 120.6, 122.9, 129.1, 136.1, 136.9, 137.4, 139.2, 151.2. Anal. Calcd for C_17_H_16_ClNO_3_: C, 64.26; H, 5.08; N, 4.41. Found: C, 64.29; H, 5.09; N, 4.45. LCMS (ESI+) *m*/*z* = 318.1 [M + H]^+^; 340.0 [M + Na]^+^; 657.1 [2M + Na]^+^.

***Synthesis of 4-allyl-2-methoxyphenyl acetate (*19*)***

NaHCO_3_ (168.0 mg, 2.0 mmol) was added to a mixture of **EU** (154 µL, 1.0 mmol) and acetic anhydride (4.0 mL) at room temperature for 12 h. The crude product was filtered under vacuum and washed with CH_2_Cl_2_ three times (5.0 mL). No further purification was required. Pale yellow oil, 89% yield. Characterization data are in agreement with the previous literature [50].

***Synthesis of 4-allyl-2-methoxyphenyl adamantane-1-carboxylate (*20*)***

TEA (279 µL, 2.0 mmol) and DMAP (12 mg, 0.1 mmol) were added to a solution of **EU** (154 µL, 1.0 mmol) in CH_2_Cl_2_ (150 mL) at 0 °C. In the same conditions, 1-adamantanecarbonyl chloride (219 mg, 1.1 mmol) was added, and the mixture was stirred at r.t. for 48 h. Then, it was diluted with CH_2_Cl_2_ (25 mL) and washed with distilled H_2_O (20 mL) three times. The combined organic layers were dried over Na_2_SO_4_, filtered, and evaporated under reduced pressure. The crude material was purified by silica gel chromatography using CHCl_3_ as the eluent. White solid, m.p. 97–98 °C, 73% yield. Characterization data are in agreement with the previous literature [51]. LCMS (ESI+) *m*/*z* = 349.2 [M + Na]^+^; 675.3 [2M + Na]^+^.

***General Procedure for the synthesis of β-phenylcalcogeno alcohols *23*–*25****

A solution of epoxide **21** (0.5 mmol) and phenylchalcogeno silane (PhSSiMe_3_, PhSeSiMe_3_, or PhTeSiMe_3_) (0.6 mmol) in dry THF (3 mL) was treated with TBAF (0.24 mL of 1 M THF solution, 0.24 mmol). The reaction was stirred at r.t. for 5 h until complete consumption of the starting material was observed by TLC. Afterward, sat. aq. NH_4_Cl was added (2 mL). The crude product was extracted with CH_2_Cl_2_ (20 mL) three times, the combined organic layers were dried over Na_2_SO_4_, filtered, and evaporated under reduced pressure to afford the crude material, which was purified by flash column chromatography using petroleum ether/EtOAc 3:1 as the eluent to afford β-phenylchalcogeno alcohols **23**–**25**.

***Synthesis of 4-[2-hydroxy-3-(phenylthio)propyl]-2-methoxyphenol (*23*)***

Yellowish oil, 75% yield. ^1^H NMR (400 MHz, CDCl_3_) *δ* 2.50 (s,1H, OH), 2.79–2.82 (m, 2H, CH_2_), 2.93 (dd, *J* 1.2, 8.0 Hz, 1H, CH_a_H_b_S), 3.11 (dd, *J* 1.2, 4.4 Hz, 1H, CH_a_H_b_S), 3.83 (s, 3H, CH_3_O), 3.90 (m, 1H, CHOH), 5.69 (s, 1H, OH), 6.67–6.71 (m, 2H), 6.84–6.86 (m, 1H), 7.20–7.35 (m, 5H). ^13^C NMR (100 MHz, CDCl_3_) *δ* 40.7, 41.9, 55.9, 70.7, 111.9, 114.5, 122.0, 126.5, 129.0, 129.4, 129.7, 135.4, 144.4, 146.5. MS (ESI+) 292.0 [M + H]^+^.

***Synthesis of 4-[2-hydroxy-3-(phenylselanyl)propyl]-2-methoxyphenol (*24*)***

Yellowish oil, 68% yield. ^1^H NMR (400 MHz, CDCl_3_) *δ* 2.84 (s, 1H, OH), 2.79–2.82 (m, 2H, CH_2_), 2.93 (dd, *J* 1.2, 8.0 Hz, 1H, CH_a_H_b_Se), 3.11 (dd, *J* 1.2, 4.4 Hz, 1H, CH_a_H_b_Se), 3.81 (s, 3H, CH_3_O), 3.90 (m, 1H, CHOH), 5.78 (s, 1H, OH), 6.63–6.68 (m, 2H), 6.83 (d, *J* 8.4 Hz,1H), 7.18–7.25 (m, 3H), 7.47–7.54 (m, 2H). ^13^C NMR (100 MHz, CDCl_3_) *δ* 35.6, 42.4, 55.8, 71.3, 111.9, 114.5, 122.0, 127.2, 129.2, 129.5, 129.5, 132.7, 144.3, 146.5. MS (ESI+) 361.2 [M + Na]^+^.

***Synthesis of 4-[2-hydroxy-3-(phenyltellanyl)propyl]-2-methoxyphenol (*25*)***

Yellowish oil, 46% yield. ^1^H NMR (400 MHz, CDCl_3_) *δ* 2.73–2.89 (m, 3H), 3.04 (dd, *J* 1.2, 7.2 Hz, 1H), 3.13 (dd, *J* 0.8, 4.0 Hz, 1H), 3.84 (s, 3H, CH_3_O), 3.96 (m, 1H, CHOH), 6.66 (d, *J* 11.6 Hz, 2H), 6.83 (d, *J* 8.0 Hz, 1H), 7.17–7.21 (m, 2H), 7.25–7.31 (m, 1H), 7.71–7.73 (m, 2H). ^13^C NMR (100 MHz, CDCl_3_) *δ* 18.4, 43.8, 55.8, 72.4, 111.4, 111.8, 114.4, 122.0, 127.7, 129.3, 129.5, 138.3, 144.4, 146.5. MS (ESI+) 389.0 [M + H]^+^.

***Synthesis of 4-[2-hydroxy-3-(pentylthio)propyl]-2-methoxyphenol (*26*)***

A solution of 1-pentanethiol (62.4 mg, 0.6 mmol) in DMF (2 mL) was cooled at 0 °C and then treated with Cs_2_CO_3_ (91 mg, 0.6 mmol) and TBAI (120 mg, 0.6 mmol). Epoxide **21** (90.0 mg, 0.5 mmol) was added, and the reaction mixture was allowed to warm to r.t. and stirred for 5 h. Afterward, the mixture was treated with saturated aqueous NH_4_Cl (2 mL) and EtOAc (5 mL) was added. The aqueous phase was extracted with EtOAc twice (5 mL), and the combined organic layers were dried over Na_2_SO_4_, filtered, and evaporated under reduced pressure to afford a crude product, which was purified on silica gel (petroleum ether/EtOAc 2:1) to afford **26** as a yellowish oil, 63% yield. ^1^H NMR (400 MHz, CDCl_3_) *δ* 0.88 (t, *J* 6.8 Hz, 3H), 1.26–1.39 (m, 3H), 1.58–1.59 (m, 3H), 2.46–2.52 (m, 4H), 2.74–2.77 (m, 3H), 3.87–3.88 (m, 4H), 5.52 (s, 1H), 6.73 (t, *J* 4.8 Hz, 2H), 6.84 (d, *J* 1.2 Hz, 1H). ^13^C NMR (100 MHz, CDCl_3_) *δ* 13.9, 22.2, 22.3, 28.9, 29.4, 30.7, 30.9, 32.4, 39.2, 39.2, 42.1, 55.9, 70.7, 111.9, 114.4, 121.9, 129.7, 144.3, 146.5. MS (ESI+) 307.1 [M + Na]^+^.

***Synthesis of 4-[3-(butylselanyl)-2-hydroxypropyl]-2-methoxyphenol (*27*)***

NaBH_4_ (38.0 mg, 1.0 mmol) was added to dibutyl diselenide (68.0 mg, 0.25 mmol) in EtOH (2 mL) and the mixture was stirred at r.t. for 15 min, during which the reddish mixture became colorless. Epoxide **21** (90 mg, 0.5 mmol) was added, and the mixture was stirred for an additional 6 h. Afterward, the mixture was treated with sat. aq. NH_4_Cl (2 mL) and EtOAc (5 mL) was added. The aqueous phase was extracted with EtOAc twice (5 mL). The combined organic layers were dried over Na_2_SO_4_, filtered, and evaporated under reduced pressure to afford a crude product, which was purified on silica gel (petroleum ether/EtOAc 2:1) to afford **27** as a yellowish oil, 92% yield. ^1^H NMR (400 MHz, CDCl_3_) *δ* 0.89 (t, *J* 7.4 Hz, 3H), 1.32–1.44 (m, 2H), 1.57–1.65 (m, 2H), 2.51–2.57 (m, 3H), 2.58 (dd, *J* 5.2, 7.7 Hz, 2H), 2.75 (dd, *J* 3.5, 9.2 Hz, 1H), 2.77 (ap d, *J* 6.5 Hz, 2H), 3.82–3.88 (m, 1H), 3.86 (s, 3H), 5.63 (bs, 1H, OH), 6.69 (dd, *J* 1.8, 8.0 Hz, 1H), 6.73 (d, *J* 1.8 Hz, 1H), 6.84 (d, *J* 8.0 Hz, 1H). ^13^C NMR (100 MHz, CDCl_3_) *δ* 13.6, 22.9, 24.5, 32.0, 32.7, 42.6, 55.9, 71.2, 111.8, 114.4, 121.9, 129.8, 144.2, 146.4. MS (ESI+) 319.2 [M + H]^+^.

***Synthesis of 4,4′-[selenobis(2-hydroxypropane-3,1-diyl)]bis(2-methoxyphenol) (*28*)***

Elemental selenium (38.0 mg, 0.5 mmol), sodium hydroxymethanesulfinate dihydrate (154.0 mg, 1.0 mmol), and NaOH (80.9 mg, 2.0 mmol) were placed in a 5 mL vial with 2 mL of H_2_O. The vial was sealed, and the reaction mixture was stirred for 30 min at r.t. under a nitrogen atmosphere. The color of the solution turned from red to orange-yellow. Then, epoxide **21** (153.0 mg, 0.85 mmol) was added and the reaction mixture was stirred at r.t. for an additional 4 h. Afterward, the mixture was treated with sat. aq. NH_4_Cl (2 mL) and EtOAc (5 mL) was added. The aqueous phase was extracted with EtOAc twice (5 mL). The combined organic layers were dried over Na_2_SO_4_, filtered, and evaporated under reduced pressure to afford a crude product, which was purified on silica gel (petroleum ether/EtOAc 2:1) to afford **28** as a yellowish oil, 63% yield. ^1^H NMR (400 MHz, CDCl_3_) *δ* 2.63 (dd, *J* 4.2, 8.1 Hz, 2H), 2.66 (dd, *J* 4.1, 8.1 Hz, 2H), 2.74–2.76 (m, 8H), 2.80–2.85 (m, 4H), 2.98 (bs, 4H, OH), 3.84 (s, 6H), 3.85 (s, 6H), 3.89–3.95 (m, 4H), 5.70 (bs, 4H, OH), 6.67 (d, *J* 8.1 Hz, 4H), 6.70 (s, 4H), 6.82 (d, *J* 8.1 Hz, 4H). ^13^C NMR (100 MHz, CDCl_3_) *δ* 14.2, 21.0, 32.4, 32.5, 42.9, 42.9, 55.9, 60.4, 72.1, 72.4, 111.9, 114.5, 121.9, 129.6, 129.6, 144.3, 146.6, 171.2. MS (ESI+) 465.2 [M + Na]^+^.

### 3.2. Anti-H. pylori Activity Testing

MIC and MBC determinations were performed by modified broth microdilution assay as previously described and the plates were examined visually after 72 h [52] on a reference strain and three clinical isolates. A control without the test molecules was also carried out. Antibiotic susceptibility was assessed according to EUCAST guidelines and MICs and MBCs are expressed in μg/mL as the average from experiments performed at least in triplicate. Antibiotic susceptibility of the strains: MTZ+ = metronidazole resistant (MIC > 8 μg/mL); MTZ− = metronidazole susceptible (MIC ≤ 8 μg/mL); CLR+ = clarithromycin resistant (MIC > 0.25 μg/mL); CLR− = clarithromycin susceptible (MIC ≤ 0.25 μg/mL); AMX+ = amoxicillin resistant (MIC > 0.125 μg/mL); AMX− = amoxicillin susceptible (MIC ≤ 0.125 μg/mL) [53].

### 3.3. In Silico Studies

All designed compounds were analyzed by means of SwissADME tool [54] and were found to not be potential pan assay interference compounds (PAINS), with the exception of the diazoderivatives due to the diazo function alert [55]. SwissAdme analysis assessed the compounds solubility by classifying them through the ESOL, Ali, and Silicos-IT classes. Herein, we have reported the former classification. Series A was found to be moderately soluble, Series B includes compounds **8**–**12** and **19** that were found to be soluble and compounds **13**–**18** and **20** that were found to be moderately soluble. In series C, intermediate **21** and compound **22** were found to be very soluble, compounds **23**–**24**, **26**–**27**, and **30** soluble, and compounds **25**, **28**, and **29** moderately soluble. In silico toxicity was assessed in silico by means of Molbook software [56] and VenomPred tool [57]. Some compounds showed some alerts and thus require further in wet investigation.

## 4. Conclusions

Despite the bioactive compounds that nature can provide, medicinal chemists’ interventions are often required in order to modify the chemical structures of the compounds in order to improve and modulate their biological function toward a biomedical purpose. In this context, **EU** is a natural compound widely distributed in essential oils that is endowed with a broad antimicrobial activity profile that also includes anti-*H. pylori* activity. In this work, we have reported our (MedChem) role in the design of three different chemical modifications of its structure. Thus, we have explored the chemical space around the phenolic OH group in its *ortho* position through aryl diazotation or derivatizing it via *O*-alkylation and *O*-benzylation, and, in the end, replacing the allyl tail, also by introducing one or more chalcogen atoms. Antibacterial susceptibility testing on four strains of *H. pylori*, including three clinical isolates, highlighted improved activity in some cases. In particular, some chalcogen-containing compounds—**26**, **27**, **29**, and **30**—displayed 2- (compounds **26**–**27** and **30**, MIC = 16 µg/mL on NCTC 11637 strain) or 4- (compound **26**, MIC = 4 µg/mL on NCTC 11637 strain) dilution-fold lower MICs compared with **EU**, while compound **28** showed worsened bioactivity (MIC = 64 µg/mL on NCTC 11637 strain).

The antibacterial activity was maintained in the resistant clinical isolates; thus, we can assume that the proposed compounds act through an innovative mechanism of action, not shared with already known antibiotics and a specific resistome that has yet to be generated. This finding is very encouraging for the further development of anti-*H. pylori* agents. Moreover, all the compounds exhibited a bactericidal behavior, which is auspicious in terms of the reduction of the risk of drug-resistant phenomena. However, an in-depth study of generations will be conducted to assess the possible rate of bacterial genome mutation after the administration of the compounds. A combination study with antibiotics currently used in clinics will also be performed in order to preserve this notable feature in future supplication and to avoid the rapid rise of resistance of the biological targets involved. The antibacterial profile of the compounds will also be investigated against different bacterial species to assess if they possess broad- or narrow-spectrum activity.

## Figures and Tables

**Table 1:** Antibacterial activity (MIC and MBC values) on four *H. pylori* strains of series A–C compounds and reference antibiotics.

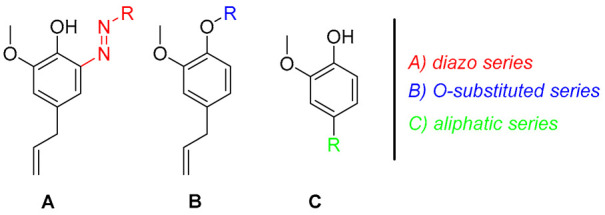			MIC/MBC (µg/mL) on *H. pylori* Strains *				MIC_50_/MIC_90_ µg/mL	MBC_90_ µg/mL
Series	CPD	R	NCTC 11637 **	F1 ***	23 ***	F40/499 ***		
**A**	**1**	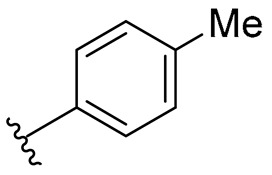	64/128	64/128	64/128	128/128	64/128	128
	**2**	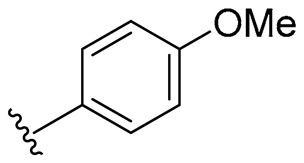	32/64	32/64	32/32	32/64	32/32	64
	**3**	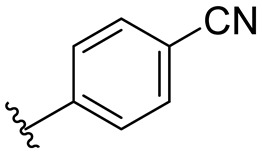	>128/>128	>128/>128	>128/>128	>128/>128	>128/>128	>128
	**4**	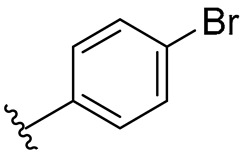	128/128	128/128	128/128	128/128	128/128	128
	**5**	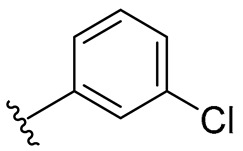	8/8	8/8	16/16	8/8	8/16	16
	**6**	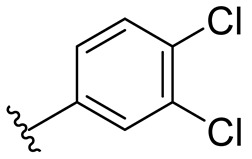	128/>128	128/128	128/>128	128/128	128/>128	>128
	**7**	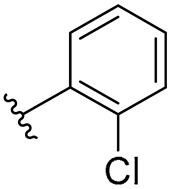	8/8	8/8	16/16	8/8	8/16	16
**B**	**8**	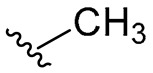	64/64	64/128	128/128	128/128	64/128	128
	**9**	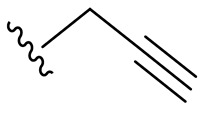	32/64	32/32	32/32	64/64	32/64	64
	**10**	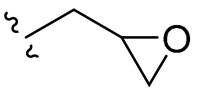	32/32	32/32	32/64	32/64	32/32	64
	**11**	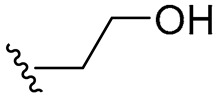	64/64	32/32	64/64	32/32	32/64	64
	**12**	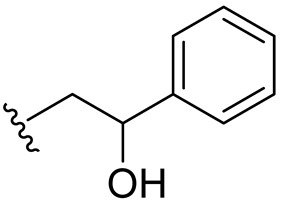	8/8	8/8	8/8	8/8	8/8	8
	**13**	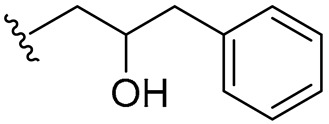	16/32	8/8	16/16	8/8	8/16	32
	**14**	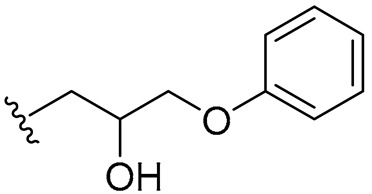	16/32	8/8	16/16	8/8	8/16	32
	**15**	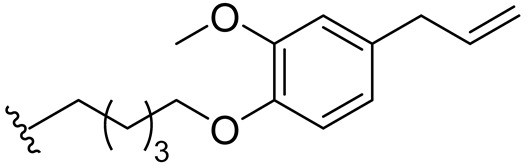	128/128	32/64	128/128	32/64	32/128	128
	**16**	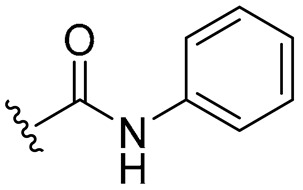	16/16	16/32	32/32	16/16	16/32	32
	**17**	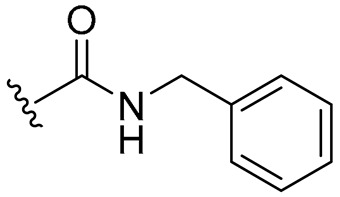	8/16	8/8	8/8	8/8	8/8	16
	**18**	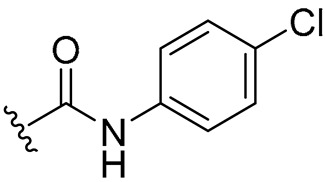	16/32	16/16	16/16	8/8	16/16	32
	**19**	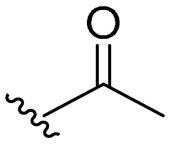	64/64	64/64	128/128	64/128	64/128	128
	**20**	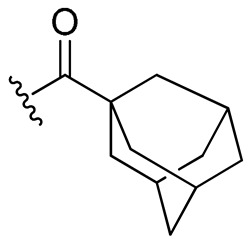	32/64	32/64	64/64	32/32	32/64	64
**C**	**21**	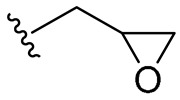	64/128	64/64	128/>128	32/32	64/128	>128
	**22**	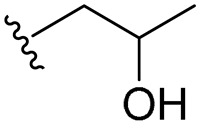	128/128	64/64	128/128	64/64	64/128	128
	**23**	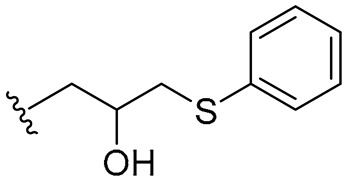	8/8	8/8	8/8	8/8	8/8	8
	**24**	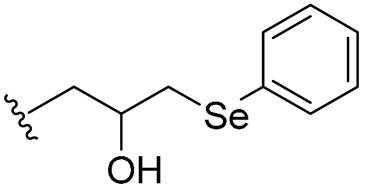	8/16	8/8	4/8	2/2	4/8	16
	**25**	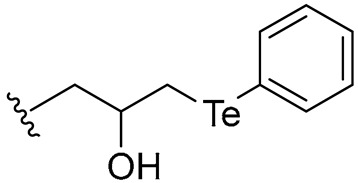	16/16	4/8	4/8	4/4	4/16	16
	**26**	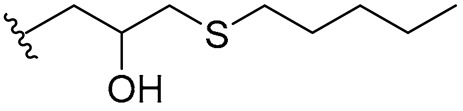	16/16	8/8	8/16	8/8	8/16	16
	**27**	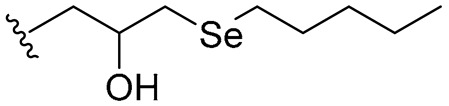	16/32	16/16	16/16	16/16	16/16	32
	**28**	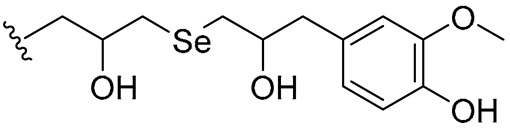	64/64	32/32	32/64	16/16	32/64	64
	**29**	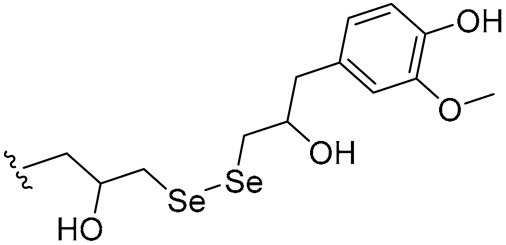	4/4	4/4	2/4	2/2	2/4	4
	**30**	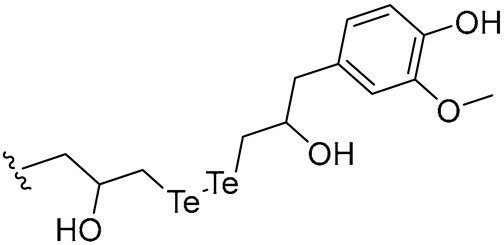	16/16	4/8	4/8	4/4	4/16	16
**EU** Parent compound			32/64	64/128	64/64	64/128	64/128	128
**Metronidazole (MTZ)**			256/256	2/2	1/1	32/32	2/256	256
**Clarithromycin (CLR)**			0.064/0.064	4/8	0.064/0.064	8/8	0.064/8	8
**Amoxicillin (AMX)**			0.016/0.016	0.064/0.064	0.016/0.016	0.016/0.032	0.016/0.064	0.064
**Antibiotic susceptibility**			MTZ+ CLR− AMX−	MTZ− CLR+ AMX−	MTZ− CLR− AMX−	MTZ+ CLR+ AMX−		

* Antibiotic susceptibility was assessed according to EUCAST guidelines (Clinical Breakpoint Tables v. 13.0, valid from 1 January 2023). MICs and MBCs are expressed in μg/mL as the average from experiments performed in triplicate; ** Reference strains; *** Clinical isolates.

## Data Availability

Data is contained within the article.
